# Laser Doppler Flare Imaging and Quantitative Thermal Thresholds Testing Performance in Small and Mixed Fiber Neuropathies

**DOI:** 10.1371/journal.pone.0165731

**Published:** 2016-11-08

**Authors:** Alon Abraham, Majed Alabdali, Abdulla Alsulaiman, Ari Breiner, Carolina Barnett, Hans D. Katzberg, Leif E. Lovblom, Bruce A. Perkins, Vera Bril

**Affiliations:** 1 Ellen and Martin Prosserman Centre for Neuromuscular Diseases, Division of Neurology, Department of Medicine, University Health Network, University of Toronto, Toronto, Canada; 2 Department of Neurology, King Fahad Hospital of the University, University of Dammam, Dammam, Saudi Arabia; 3 Division of Endocrinology and Metabolism, Department of Medicine, Mount Sinai Hospital and Lunenfeld Tanenbaum Research Institute, University of Toronto, Toronto, Canada; Hirosaki Daigaku, JAPAN

## Abstract

**Introduction:**

Small fiber neuropathy might be a part of typical mixed small and large fiber neuropathy, or a distinct entity, affecting exclusively small nerve fibers.

**Objectives:**

Explore the utility of small nerve fiber testing in patients with clinical presentation suggesting small fiber neuropathy, with and without evidence for concomitant large fiber neuropathy.

**Methods:**

Patients attending the neuromuscular clinic from 2012 to 2015 with a clinical presentation suggesting small nerve fiber impairment, who had Laser Doppler flare imaging (LDI_Flare_) and quantitative thermal testing (QTT) were evaluated for this study. Patients with clinical or electrophysiological evidence for concomitant large fiber neuropathy were not excluded.

**Results:**

The sensitivities of LDI_Flare_, cooling and heat threshold testing were 64%, 36%, and 0% respectively for clinically highly suggestive small fiber neuropathy, 64%, 56%, and 19% respectively for mixed fiber neuropathy, and 86%, 79%, and 29% respectively for diabetic mixed fiber neuropathy.

**Discussion:**

LDI_Flare_ and cooling thresholds testing are non-invasive small nerve fiber testing modalities, with moderate performance in patients with small and mixed fiber neuropathy, and excellent performance in diabetic mixed fiber neuropathy.

## Introduction

Small fiber neuropathy might be a part of typical common mixed fiber neuropathy, involving large and small nerve fibers concomitantly, or a distinct entity, affecting exclusively small nerve fibers[1]. Typically, small fiber neuropathy presents with positive sensory symptoms such as burning pain and allodynia, occasionally accompanied by reduced distal pinprick and temperature perception on the neurological examination, which is otherwise normal. Diagnosis is made on the basis of the clinical features, normal nerve conduction studies, and confirmed by specialized tests of small-fiber function[2]. Due to their physiologic characteristics, small nerve fibres cannot be investigated by routine electrophysiological studies, making the diagnosis particularly difficult[1]. Skin biopsy has become the pathologic gold standard used for the diagnosis of a small fiber neuropathy, considered to be a reliable and efficient technique[3], however, it is invasive and costly.

Laser Doppler flare imaging (LDI_Flare_), and quantitative thermal testing (QTT) are non-invasive small fiber testing modalities, measuring small nerve fiber function, enabling objective confirmation of small fiber neuropathy. Nonetheless, they may be utilized also in mixed fiber neuropathies, as in diabetic sensorimotor peripheral neuropathy[4,5]. In axon reflex-mediated neurogenic vasodilatation (LDI_Flare_), a heat stimulus is used, activating peripheral C-fiber branches in the surrounding skin. As a result, focal increase in blood flow occurs, measured by a scanning Doppler infrared laser beam. LDI_Flare_ was shown to be reproducible and useful, correlating with nerve fiber density[6]. QTT of cooling and thermal thresholds is increasingly used for the evaluation of peripheral nervous system function in the clinical and research domains, mainly for confirmation of small nerve fiber neuropathy, and has been found to be reliable and reproducible[7]. Both of these methods are non-invasive, and can be performed at the bedside with immediate results at a reasonable cost. In this study, we aimed to explore the utility of these small nerve fiber testing modalities in patients with symptoms suggestive for small nerve fiber impairment, not excluding those with clinical or electrophysiological evidence for mixed fiber neuropathy, regarding sensitivity and correlations with clinical and electrophysiological characteristics.

## Materials and Methods

Patients attending the neuromuscular clinic from 2012 to 2015 with suspected small fiber neuropathy, performing small nerve fiber testing using LDI_Flare_, QTT and corneal confocal microscopy (CCM) testing, were evaluated for this study. CCM testing findings will be reported separately.

Small fiber testing was performed in 3 different groups of patients. All patients had a clinical presentation suggesting small fiber neuropathy, typically with positive sensory symptoms such as burning pain and allodynia, and normal or close to normal neurological examination, excluding decreased temperature and pinprick sensation. The first group included patients with clinically highly suggestive small fiber neuropathy, defined by sensory symptoms restricted to the lower limbs, with no associated weakness, in the presence of normal neurological examination other than decreased temperature and pinprick sensation, and normal NCS. The second group included patients with clinically suspected small fiber neuropathy, not fulfilling completely the previously described definition for highly suggestive small fiber neuropathy, but also with normal NCS. The third group included patients with mixed fiber neuropathy, with abnormal NCS, defined for the purpose of this study a sural sensory nerve action potential (SNAP) below 7 μV, which is the threshold used by our laboratory. This patient group was referred to our clinic specifically for small fiber testing, and NCS done subsequently were found to be abnormal.

In this retrospective study, we extracted demographic data, clinical history, neurological and electrophysiological examinations results, as well as vibration perception thresholds (VPT) and small fiber testing results using LDI_Flare_ and QTT testing. The Research Ethics Board of the University Health Network approved the current study protocol and waived informed consent.

Nerve conduction studies (NCS) were performed using the Sierra Wave instrument (Cadwell Laboratories Inc., Kennewick, WA, USA). Age- and height-adjusted NCS reference values were used, according to the standards of the Toronto General Hospital (University Health Network) electrophysiology laboratory. Limb temperature was measured prior to nerve conduction studies, and if required, warming was performed to ensure a surface temperature of ≥32.0°C in the hands and ≥31.0°C in the feet.

Peroneal and sural NCS were performed using surface stimulating and recording techniques according to the standards of the Canadian Society of Clinical Neurophysiology and the American Association of Neuromuscular and Electrodiagnostic Medicine[8]. The electromyography Instrument calculated latencies, amplitudes and conduction velocities automatically. Peroneal nerve compound motor action potential (CMAP) amplitudes and Sural SNAP amplitudes were measured from first negative peak to the next positive peak.

LDI_Flare_ technique was used to measure heat induced axon reflex-mediated neurogenic vasodilation, which might be influenced by sympathetic activity and basal levels of nitric oxide[9]. Temperature on the dorsum foot temperature was standardized to 32°C using a warm blanket for at least 20 minutes. Subsequently, a skin-heating probe (Moor Instruments Ltd, Axminster, U.K.) was used to heat the skin above the first metatarsal area on the dorsum of the foot to 44°C for 20 minutes. The LDI apparatus used a scanning Doppler infrared laser beam with a wavelength of 785 nm, sufficient to penetrate skin to register the movement of blood cells in dermal capillaries. A 36 cm^2^ area represented a 256x256 pixel resolution with each pixel itself representing a measurement of the velocity of tissue blood flow. The total scanning time was less than five minutes per examination. The MoorLDI software (version 3.11) was used to measure blood flow in the dermal capillaries and the LDI_Flare_ area was calculated in centimeters squared. Abnormal LDI_Flare_ was defined < 2 cm^2^ based on local laboratory normative data[4,10].

Cooling and heat detection thresholds were tested using a method of limits with the TSA-II NeuroSensory Analyzer (Medoc Advanced Medical Systems, Ramat-Yishai, Israel). A stimulator with a temperature of 32°C was applied to the dorsum of the foot and hand. The temperature was gradually decreased to the first level detected by the patient as a cooler than the preceding for cooling threshold testing, and gradually increased to the first level detected by the patient as a warmer than the preceding for heat threshold testing. An average of the five levels was taken for each of the studies on the foot and hand, and compared to age-matched normative data. A catch trial, with null stimulus, was inserted randomly during testing.

VPT testing was performed with a Neurothesiometer, using the method of limits[11]. The stimulus was applied to the distal pulp of the toe on each side, and the patient was requested to indicate when vibration sensation was first perceived. Stimulus strength was gradually increased from null intensity to a value in voltage at which the subject first detected vibration. Testing was carried out with the subject’s eyes closed. Three separate tests were conducted, and a mean of the three values was calculated in volts. A ‘null stimulus’ trial was added randomly to ensure the subject’s adherence and understanding. Testing generally required less than 3 min. Normal values were considered 15 or less in the toes.

Skin biopsy was performed 10 cm proximal to the lateral malleolus using a 3 mm circular punch, at a depth of 4 mm, after cleaning the skin with an alcohol swab and anaesthetizing the skin with 2% lidocaine with epinephrine.

### Statistical analysis

Statistical analysis was performed using SAS version 9.2 for Windows (SAS Institute, Cary, North Carolina). Baseline participant characteristics were expressed as means ± standard deviations (SD) for continuous data, or as frequency and percentage for categorical data. Continuous data was assessed for normality (Shapiro-Wilk). For each small fibre test, results were dichotomized into normal and abnormal based on reference values, and differences in characteristics between normal and abnormal participants were assessed using the student’s t-test, the Wilcoxon rank-sum test, or the χ^2^-test (depending on the type and distribution of the variable). Cohen’s kappa coefficient was used to determine agreement among the dichotomized small fiber tests. Pearson correlation coefficients between LDI_Flare_ area values and clinical and electrophysiological characteristics were calculated. The Benjamini-Hochberg procedure was used to adjust for multiple comparisons between the three different dichotomizations of normal and abnormal small fiber test results in the following categories of variables: abnormal examination findings, nerve conduction studies, and small fiber tests and VPT. Due to the exploratory nature of this study, the false discovery rate for this procedure was set at 0.10, otherwise, significance was set at α-level of 0.05.

## Results

The total cohort included 123 patients (S1 Dataset) with mean age of 55 ± 16 years, 61% of them were females. Common comorbidities included hypertension (40%), diabetes mellitus (26%), thyroid disease (19%), and hyperlipidemia (18%). 88% of patients had sensory symptoms in the lower limbs, and 38% had sensory symptoms in the upper limbs. On examination, muscle weakness was detected in 11%. Sensory deficits were more common for small nerve fiber modalities, including pinprick in 61% and temperature in 69%. 84% of patients had normal ankle reflexes. Electrophysiological testing showed normal mean values of amplitudes and of conduction velocities in peroneal and sural nerves in the total cohort, but abnormal nerve conduction studies, with abnormal sural nerve response were observed in 32% (Table 1).

**Table 1:** Clinical and electrophysiological characteristics, and additional tests results in total cohort, and compared between patients with normal and reduced LDI_Flare_ area values.

	Total Cohort	Normal LDI_Flare_	Reduced LDI_Flare_	p
Number	123	62	57	
Age (years)	55 ± 16	52 ± 16	58 ± 14	**<0.05**
Female	61%	71%	52%	**<0.05**
Diabetes Mellitus	26%	16%	37%	**<0.01**
Duration (years)	12 ± 12	6 ± 4	12 ± 11	**<0.05**
Hypertension	40%	36%	43%	NS
Hyperlipidemia	18%	16%	19%	NS
Thyroid disease	19%	21%	15%	NS
**Sensory symptoms**				
Upper limbs	38%	53%	23%	**<0.01**
Lower limbs	88%	92%	88%	NS
**Abnormal examination findings**				
Weakness	11%	5%	19%	**<0.05**
Vibration	47%	36%	60%	**<0.05**
Proprioception	19%	13%	27%	NS
Light touch	42%	37%	49%	NS
Pinprick	61%	52%	74%	**<0.05**
Temperature	69%	65%	76%	NS
Ankle reflex	16%	7%	25%	**<0.01**
**Nerve Conduction Studies**				
Sural nerve Amp (μV)	13 ± 10	15 ± 10	10 ± 9	**<0.01**
Sural nerve CV (m/s)	47 ± 6	49 ± 5	46 ± 6	**<0.01**
Peroneal nerve Amp (mv)	7 ± 4	8 ± 3	6 ± 4	**<0.01**
Peroneal nerve CV (m/s)	45 ± 5	47 ± 5	42 ± 5	**<0.01**
**Additional tests results**				
Cooling (elevated thresholds)	38%	13%	65%	**<0.01**
Heat (elevated thresholds)	8%	3%	14%	**<0.05**
LDI mean area (mm^2^)	2.4 ± 1.23	3.11 ± 1.37	1.66 ± 0.21	**<0.01**
LDI area range (mm^2^)	1.1–8	2–8	1.1–2	**<0.01**
VPT (V)	14 ± 8	11 ± 8	17 ± 8	**<0.01**

LDIFlare—Laser Doppler Imaging Flare; NS—Non Significant; Amp—Amplitude; CV—Conduction Velocity; VPT—Vibration Perception Thresholds.

LDI_Flare_ testing was performed in 119 patients, and QTT using cooling and heat thresholds testing in 120. 48% had reduced LDI_Flare_, 38% had elevated cooling thresholds, whereas less than 10% had abnormal heat thresholds. Reduced LDI_Flare_ and elevated cooling thresholds were associated with older age (58 years vs. 52 years), higher risk for diabetes (37% vs. 16%, and 37% vs. 19% respectively), with longer diabetes duration (12 years vs. 6 years and 14 years vs. 5 years respectively), and less frequent upper limb symptoms (23% vs. 53%, and 24% vs. 46%), compared to patients with normal studies. Patients with abnormal LDI_Flare_ and QTT had more frequent weakness, sensory loss for different sensory modalities (vibration, proprioception, light touch, pinprick and temperature perception), absent ankle reflexes, reduced sensory and motor amplitudes and conduction velocities in the lower limbs, and elevated VPT. However, these findings were more obvious in patients with abnormal LDI_Flare_ and elevated cooling thresholds, than for patients with elevated heat thresholds. Patients with abnormal LDI_Flare_ were more likely to have elevated cooling and heat thresholds, and vice versa (Tables 1 and 2).

**Table 2:** Comparison of clinical and electrophysiological characteristics, and additional tests results between patients with normal and elevated cooling and heat thresholds.

	Cooling Thresholds			Heat Thresholds		
	Normal	Elevated	p	Normal	Elevated	p
Number	75	45		110	10	
Age (years)	52 ± 17	58 ± 14	**<0.05**	55 ± 16	55 ± 11	NS
Female	61%	63%	NS	64%	33%	NS
Diabetes Mellitus	19%	37%	**<0.05**	22%	67%	**<0.01**
Duration (years)	5 ± 4	14 ± 11	**<0.01**	9 ± 10	15 ± 4	NS
Hypertension	36%	45%	NS	39%	44%	NS
Hyperlipidemia	9%	32%	**<0.01**	16%	33%	NS
Thyroid disease	21%	17%	NS	20%	11%	NS
**Sensory symptoms**						
Upper limbs	46%	24%	**<0.05**	37%	44%	NS
Lower limbs	89%	87%	NS	88%	100%	NS
**Abnormal examination findings**						
Weakness	6%	21%	**<0.05**	11%	20%	NS
Vibration	29%	74%	**<0.01**	42%	89%	**<0.05**
Proprioception	12%	34%	**<0.01**	16%	44%	**<0.05**
Light touch	29%	63%	**<0.01**	40%	67%	NS
Pinprick	49%	82%	**<0.01**	60%	80%	NS
Temperature	59%	86%	**<0.01**	68%	90%	NS
Ankle reflex	4%	33%	**<0.01**	13%	40%	**<0.05**
**Nerve Conduction Studies**						
Sural nerve Amp (μV)	15 ± 11	8 ± 6	**<0.01**	13 ± 10	5 ± 6	**<0.01**
Sural nerve CV (m/s)	48 ± 6	46 ± 6	NS	48 ± 6	44 ± 10	NS
Peroneal nerve Amp (mv)	8 ± 3	6 ± 4	**<0.05**	7 ± 3	5 ± 4	**<0.05**
Peroneal nerve CV (m/s)	46 ± 5	42 ± 5	**<0.01**	45 ± 5	42 ± 7	NS
**Additional tests results**						
Cooling (elevated thresholds)				33%	90%	**<0.01**
Heat (elevated thresholds)	1.3%	20%	**<0.01**			
LDI_Flare_ (reduced area)	27%	82%	**<0.01**	45%	80%	**<0.05**
VPT (V)	12 ± 6	18 ± 9	**<0.01**	13 ± 7	20 ± 12	**<0.01**

LDIFlare—Laser Doppler Imaging Flare; NS—Non Significant; Amp—Amplitude; CV—Conduction Velocity; VPT—Vibration Perception Thresholds.

Sensitivities for patients with clinically highly suggestive small fiber neuropathy for LDI_Flare,_ cooling and heat thresholds testing, were 64%, 36% and 0% respectively (Table 3).

**Table 3:** The clinical and electrophysiological characteristics, and sensitivities of LDI_Flare_, cooling and heat thresholds for diagnosing small and mixed fiber neuropathy in different neuropathy groups.

	Small Fiber*	Mixed**	DM	Mixed + DM
Number	11	36	30	14
Age (years)	44 ± 18	60 ± 16	55 ± 14	55 ± 16
Female	67%	47%	43%	46%
Diabetes Mellitus	11%	37%	100%	100%
Duration (years)	4	11 ± 11	12 ± 12	11 ± 11
IGT	0%	9%	-	-
Hypertension	0%	59%	56%	75%
Hyperlipidemia	22%	24%	31%	45%
Thyroid disease	11%	24%	23%	27%
**Sensory symptoms**				
Upper limbs	0%	19%	26%	17%
Lower limbs	100%	94%	93%	92%
**Abnormal examination findings**				
Weakness	0%	9%	17%	15%
Vibration	0%	79%	62%	79%
Proprioception	0%	37%	23%	45%
Light touch	0%	64%	74%	69%
Pinprick	25%	77%	86%	100%
Temperature	44%	82%	93%	93%
Ankle reflex	0%	32%	34%	62%
**Nerve Conduction Studies**				
Sural Amp (μV)	16 ± 5	4 ± 2	11 ± 9	4 ± 3
Sural CV (m/s)	49 ± 4	45 ± 6	46 ± 6	46 ± 6
Peroneal Amp (mv)	8 ± 2	5 ± 3	7 ± 4	6 ± 4
Peroneal CV (m/s)	44 ± 5	42 ± 5	42 ± 5	40 ± 6
**Small fiber tests sensitivities**				
LDI_Flare_	(7/11) 64%	(23/36) 64%	(20/29) 69%	(12/14) 86%
Cooling	(4/11) 36%	(20/36) 56%	16/29 (55%)	(11/14) 79%
Heat	(0/11) 0%	(7/36) 19%	6/29 (21%)	(4/14) 29%
Combined	(7/11) 64%	(26/36) 72%	(22/29) 76%	(14/14) 100%

* Small Fiber—Clinically highly suggestive small fiber neuropathy, in the presence of normal NCS;

** Mixed fiber—Clinical presentation suggesting small fiber impairment, in the presence of abnormal NCS;

LDIFlare—Laser Doppler Imaging Flare; IGT—Impaired gloucose tolerance; Amp—Amplitude; CV—Conduction velocity.

Intraepidermal nerve fiber density (IENFD) testing showed reduced nerve fiber density in 3 out of 3 patients, who also had reduced LDI_Flare_.

The sensitivities for the detection of mixed fiber neuropathy with low sural SNAP amplitude, for LDI_Flare,_ cooling and heat thresholds testing were 64%, 56%, and 19% respectively. In patients with diabetes sensitivities were 69%, 55%, and 21% respectively. In patients with the combination of diabetes and low sural SNAP amplitude, higher sensitivities were found, of 86%, 79%, and 29% respectively, with 100% sensitivity for the combination of these tests.

Agreement between the different small fiber testing modalities was good (k = 0.52, p<0.0001) between LDI_Flare_ and cooling thresholds testing, but marginal (k = 0.11, p = 0.02) between LDI_Flare_ and heat thresholds testing, as well as between cooling and heat thresholds testing (k = 0.22, p = 0.0002).

There was statistically significant correlation between LDI_Flare_ area values and sensory and motor conduction velocities, and VPT at the toes (Table 4).

**Table 4:** Pearson correlation coefficients between LDI_Flare_ area values and clinical and electrophysiological characteristics.

Comparison measure	Pearson r	p Value
Age (years)	-0.13	0.20
Diabetes duration (years)	0.25	0.23
**Nerve Conduction Studies**		
Sural nerve Amp (μV)	0.06	0.56
Sural nerve CV (m/s)	0.27	**0.0045**
Peroneal nerve Amp (mv)	0.13	0.20
Peroneal nerve CV (m/s)	0.35	**0.0002**
**Additional tests results**		
VPT finger (V)	-0.17	0.10
VPT toe (V)	-0.33	**0.0011**

LDIFlare—Laser Doppler Imaging Flare; Amp—Amplitude; CV—Conduction Velocity; VPT—Vibration Perception Thresholds.

## Discussion

Our study results show that abnormal LDI_Flare_ and QTT testing can provide objective confirmation for the presence of small fiber neuropathy, with a moderate combined sensitivity of 64%. We found clear superiority for LDI_Flare_ testing, showing similar sensitivity of 64%, whereas QTT had much lower sensitivities (36% for cooling and 0% for heat threshold testing), not adding to the overall sensitivity (Table 3). Slightly higher combined sensitivity of 72% was demonstrated in patients with mixed fiber neuropathy, with clinical presentation suggesting small fiber neuropathy in the presence of abnormal NCS. Interestingly, sensitivity was high in the subgroup of diabetic patients with mixed fiber neuropathy, reaching 86% for LDI_Flare_, 79% for cooling and 29% for heat threshold testing. Combined together, they were found to be 100% sensitive for this patient subgroup. The high yield of these testing in diabetic patients with mixed fiber neuropathy might be explained by frequent small nerve fiber impairment in this particular patient subgroup, which fits the common clinical impression.

The association of abnormal LDI_Flare_ and QTT testing with mixed fiber neuropathy, as reflected by the clinical and electrophysiological examinations, is in agreement with previous studies[4,5,12–14]. Patients with abnormal small fiber testing in our cohort, had more frequent limb muscle weakness, reduced vibration and proprioception perception, and absent ankle reflexes on the neurological examination, indicating concomitant large fiber impairment. This was confirmed by NCS, showing lower sensory and motor nerve amplitudes and conduction velocities (Tables 1 and 2), and significant linear correlation between LDI_Flare_ area values and sensory and motor conduction velocities, and VPT (Table 4). The higher frequency of large fiber impairment in patients with abnormal small fiber testing, might suggest in turn a correlation between the degree of large fiber impairment and the presence of small fiber impairment. Similarly, in a previous study of 74 consecutive patients with small fiber neuropathy, patients with reduced IENFD had relatively lower sural sensory amplitudes. Although values still fell within the normal range, concomitant early large fiber impairment could not be excluded. However, in this study similar correlations between abnormal small fiber testing and sensory deficits for different modalities were not found.[10] Nonetheless, reduced pinprick sensation has been reported in patients with reduced IENFD[15]. These findings might suggest that there is a spectrum of small and large fiber neuropathies, with mixed nerve fiber neuropathy being the rule.

Patients with abnormal LDI_Flare_ and QTT testing were older, and had a twofold risk for diabetes. These findings are not surprising, considering the known decrease in small fiber density with age,[16] and the fact that diabetes is the most common cause for small and mixed fiber neuropathy. In contrast, patients with normal LDI_Flare_ and QTT testing, had a twofold higher frequency of upper limb sensory symptoms, which although known to exist in certain forms of small fiber neuropathy[2] and other common neuromuscular disorders (such as carpel tunnel syndrome, cervical radiculopathies, etc.), is less typical for common peripheral neuropathy. As expected, patients with abnormal LDI_Flare_ and QTT testing had a higher frequency of abnormal pinprick and temperature sensation, which is typical for small fiber neuropathy.

Among small fiber testing performed, heat threshold testing was found to be relatively inferior to cold threshold testing and LDI_Flare._ Elevated heat thresholds were found in less than 10% of patients, whereas LDI_Flare_ and cold threshold testing showed abnormalities in more than 40% on average. Moreover, abnormal heat threshold testing correlated less well with clinical and electrophysiological evidence for large fiber impairment, compared with LDI_Flare_ and cold threshold testing (Tables 1 and 2). Although abnormal LDI_Flare_ results were correlated with abnormal QTT and vice versa, good agreement was shown for LDI_Flare_ and cooling thresholds testing, while only marginal agreement with heat threshold testing. This might suggest that heat threshold testing is a less sensitive indicator for peripheral neuropathy, compared to its counterparts. The relative superiority of LDI_Flare_ compared to QTT testing, and better performance of cold threshold compared to heat threshold testing, has been described previously[1].

Our study has a few limitations. Our retrospective cohort explored the performance of small fiber testing, which included LDI_Flare_ and QTT testing. Although LDI_Flare_ and cooling threshold testing showed satisfactory performance, the sensitivity of heat threshold testing was found to be lower than expected compared with previous literature. We have not used additional non-invasive small nerve fiber testing modalities such as the Quantitative Sudomotor Axon Reflex Test (QSART). In addition, we did not perform IENFD testing, which is currently considered to be the gold standard for small fiber neuropathy detection, in most patients. Other non-invasive small nerve fiber testing modalities, especially while combined[17], and IENFD testing[1,18], might have slightly higher sensitivities for small fiber neuropathy detection. In addition, although the total cohort number was reasonable, the number of patients with exclusive small fiber neuropathy, and patients with diabetic mixed fiber neuropathy, was relatively low. Finally, small fiber testing sensitivities might have been skewed in a positive direction in patients with mixed fiber neuropathy, as their clinical presentation suggested small fiber impairment, leading to small fiber testing.

In conclusion, LDI_Flare_ and cooling threshold testing are non-invasive small nerve fiber testing modalities, which can be performed at the bedside with immediate results. Their sensitivities are moderate for small and mixed fiber neuropathy detection, and high for diabetic mixed fiber neuropathy. LDI_Flare_ seems to be superior to QTT, and heat threshold testing seems to be the least efficient test. Additional studies, confirming these findings and comparing these tests with additional non-invasive small fiber and IENFD testing, are warranted.

## Supporting Information

S1 Dataset (XLSX)

## Abbreviations

LDI_Flare_: Laser Doppler flare imaging
QTT: quantitative thermal testing
CCM: Corneal confocal microscopy
VPT: Vibration perception thresholds
NCS: Nerve conduction studies
CMAP: Compound motor action potential
SNAP: Sensory nerve action potential
SD: Standard deviations
IENFD: Intraepidermal nerve fiber density
